# High rates of multidrug-resistant and rifampicin-resistant tuberculosis among re-treatment cases: where do they come from?

**DOI:** 10.1186/s12879-016-2171-1

**Published:** 2017-01-06

**Authors:** Romain Ragonnet, James M. Trauer, Justin T. Denholm, Ben J. Marais, Emma S. McBryde

**Affiliations:** 1 Faculty of Medicine, Dentistry and Health Sciences, University of Melbourne, Melbourne, Australia; 2 Centre for Population Health, Burnet Institute, 85 Commercial Road, Melbourne, 3141 VIC Australia; 3 Victorian Tuberculosis Program, Melbourne Health, Melbourne, Australia; 4 School of Public Health and Preventive Medicine, Monash University, Melbourne, Australia; 5 Department of Microbiology and Immunology, University of Melbourne at the Peter Doherty Institute, Melbourne, Australia; 6 Victorian Infectious Diseases Service, Royal Melbourne Hospital, Parkville, VIC Australia; 7 Marie Bashir Institute and the Centre for Research Excellence in Tuberculosis, University of Sydney, Sydney, Australia; 8 Australian Institute of Tropical Health and Medicine, James Cook University, Townsville, Australia

**Keywords:** Tuberculosis, Multidrug-resistant tuberculosis, Re-treatment, Causal pathway, Misdiagnosis, Inappropriate therapy, Drug resistance amplification

## Abstract

**Background:**

Globally 3.9% of new and 21% of re-treatment tuberculosis (TB) cases are multidrug-resistant or rifampicin-resistant (MDR/RR), which is often interpreted as evidence that drug resistance results mainly from poor treatment adherence. This study aims to assess the respective contributions of the different causal pathways leading to MDR/RR-TB at re-treatment.

**Methods:**

We use a simple mathematical model to simulate progression between the different stages of disease and treatment for patients diagnosed with TB. The model is parameterised using region and country-specific TB disease burden data reported by the World Health Organization (WHO). The contributions of four separate causal pathways to MDR/RR-TB among re-treatment cases are estimated: I) initial drug-susceptible TB with resistance amplification during treatment; II) initial MDR/RR-TB inappropriately treated as drug-susceptible TB; III) MDR/RR-TB relapse despite appropriate treatment; and IV) re-infection with MDR/RR-TB.

**Results:**

At the global level, Pathways I, II, III and IV contribute 38% (28–49, 95% Simulation Interval), 44% (36–52, 95% SI), 6% (5–7, 95% SI) and 12% (7–19, 95% SI) respectively to the burden of MDR/RR-TB among re–treatment cases. Pathway II is dominant in the Western Pacific (74%; 67–80 95% SI), Eastern Mediterranean (68%; 60–74 95% SI) and European (53%; 48–59 95% SI) regions, while Pathway I makes the greatest contribution in the American (53%; 40–66 95% SI), African (43%; 28–61 95% SI) and South-East Asian (50%; 40–59 95% SI) regions.

**Conclusions:**

Globally, failure to diagnose MDR/RR-TB at first presentation is the leading cause of the high proportion of MDR/RR-TB among re-treatment cases. These findings highlight the need for contextualised solutions to limit the impact and spread of MDR/RR-TB.

**Electronic supplementary material:**

The online version of this article (doi:10.1186/s12879-016-2171-1) contains supplementary material, which is available to authorized users.

## Background

Multidrug-resistant tuberculosis (MDR-TB), defined as resistance to at least rifampicin and isoniazid, is a major threat to global tuberculosis (TB) control [1]. The World Health Organization (WHO) estimates that 3.9% of all new TB cases had MDR-TB or rifampicin-resistant TB (RR-TB) in 2015 [2]; in comparison to 21% of TB patients with a history of prior treatment. The dramatic gap between these two estimates can be explained by the potential for a TB patient to acquire drug resistance during treatment, particularly if there is treatment interruption or default [3–7]. However, in addition to acquired (secondary) drug resistance resulting from poor treatment adherence, multiple other factors may contribute to the higher rate of MDR/RR-TB observed among re-treatment cases.

The potential for primary transmission of drug-resistant TB has long been under-recognised [8]. A large MDR-TB outbreak in New York City [9], and a cluster of extensively drug-resistant (XDR) TB cases in South Africa provided stark evidence that some drug-resistant strains are highly transmissible [10]. Epidemic spread of MDR-TB has since been confirmed in multiple settings [11], and molecular methods have demonstrated the importance of re-infection to TB recurrence, especially in endemic areas [12]. Among clinical MDR-TB strains, the fitness cost associated with acquired drug resistance can be overcome by various compensatory mechanisms [13], and the opportunity for compensatory evolution is enhanced by selective pressure from poorly targeted treatment [14].

Although the End TB strategy calls for universal access to drug susceptibility testing (DST) [15], new TB cases remain infrequently tested for drug resistance globally. Therefore, newly presenting MDR/RR-TB patients will often receive the same regimen as drug-susceptible TB (DS-TB) cases. However, although some clinical response is possible, cure rates resulting from standard first-line treatment are low even with adequate treatment adherence and these patients mostly re-present as failure or relapse cases [16]. Given the high rate of drug resistance among re-treatment cases, they are usually prioritised for phenotypic DST or genotypic testing with Xpert MTB/RIF®, which introduces a strong case detection bias. Therefore, re-treatment cases with MDR/RR-TB represent a mixed bag, including secondary acquired and primary transmitted MDR/RR-TB. The contribution of these subgroups to the total burden of MDR/RR-TB among re-treatment cases has been poorly quantified and may be highly setting-dependent. A recent modelling study suggested that the emergence of MDR-TB is mostly driven by transmission of such strains [17], but the contribution of different causal pathways to MDR/RR-TB at re-treatment and a detailed geographic breakdown of their contribution have never been conducted. Better quantification of the contributing pathways (causes) would therefore provide insight into the evolution of the global MDR/RR-TB epidemic and guide region-specific programmatic approaches to its control.

We present a simple probability tree model, using regional disease estimates, to quantify the proportions of MDR/RR-TB at re-treatment attributed to four principle causal pathways: I) initial drug-susceptible TB with resistance amplification during treatment; II) initial MDR/RR-TB inappropriately treated as drug-susceptible TB; III) MDR/RR-TB relapse despite appropriate treatment; and IV) re-infection with MDR/RR-TB.

## Methods

### Study design

We developed a model to quantify the contribution of different causal pathways towards the high burden of MDR/RR-TB observed among re-treatment cases in 2015. Patients were not involved in this study as only data from the WHO and from published literature were used. Model parameters include key characteristics of the different WHO regions, TB burden estimates and reported data from National TB control programs for 2013. All seven WHO regions were considered: Africa (AFR), the Americas (AMR), Europe (EUR), South East Asia (SEAR), the Eastern Mediterranean (EMR) and Western Pacific (WPR) regions, as well as combined Global estimates (GLOBAL). Since the model is intended for moderate to high burden countries only, where TB transmission remains poorly controlled, a country-specific analysis was performed on the 104 countries with an estimated TB-incidence ≥50 new cases/100,000/year in 2013.

### Model principle

**Fig. 1:**
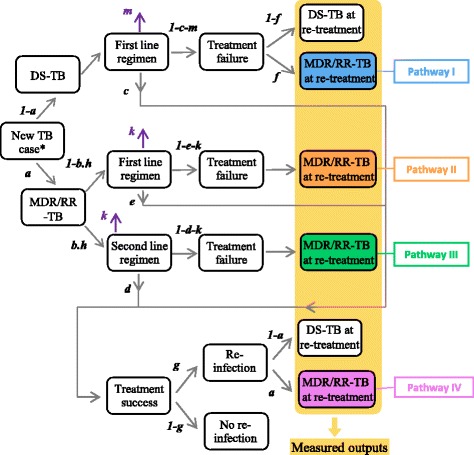
Presentation of the model structure and parameters. Parameters correspond to the probability of a patient transitioning to the state at the end of the corresponding arrow if initially in the state at the start of this arrow. The coloured boxes correspond to the outputs that we observe for quantifying the respective contributions of the different pathways to MDR/RR-TB at re-treatment. *‘new TB case’ stands for a patient presenting primary TB disease and who undergoes therapy against TB. Parameter *a* is the rate of MDR/RR-TB among new TB-cases. Parameter *b* is the DST coverage in new TB-cases while parameter *h* stands for the proportion of notified MDR/RR-TB cases that start on second-line regimen. Parameters *c* and *d* are the treatment success rates for new DS-TB cases and new MDR/RR-TB cases respectively. Parameter *e* represents the treatment success rate for MDR/RR-TB treated with first-line regimen. Parameter *f* is the risk of drug-resistance amplification for a DS-TB patient failing therapy. Parameter *g* is the proportion of recovered individual who get re-infected with TB. Parameters *m* and *k* are the death rate during treatment for DS-TB and MDR/RR-TB patients respectively

A simple probability tree model was used to simulate progression between the different stages of infection, disease and treatment for patients diagnosed with TB (Fig. 1). The use of two distinct pathways based on initial susceptibility of primary TB infection allowed consideration of parameters that are specific to DS-TB and MDR/RR-TB. For simplicity, individuals presenting with mono-resistant or poly-resistant TB without rifampicin resistance were considered to be DS-TB, while resistance beyond MDR/RR-TB was considered to be MDR/RR-TB. The model considered that new MDR/RR-TB cases were treated appropriately only if they were diagnosed as MDR/RR-TB cases and started on a second-line regimen. Accordingly, the probability that a new MDR/RR-TB case is treated appropriately is obtained by multiplying the DST coverage (*b*) by the proportion of notified MDR/RR-TB cases that start on second line regimen (*h*). Any treatment regimen could result in cure, failure (unsuccessful treatment) or death. Individuals lost to follow-up are assumed to experience failure in the baseline analysis but alternate scenarios were considered in a sensitivity analysis. Cured individuals are assumed to have cleared infection and could only be affected by a new episode of TB in case of reinfection.

Only DS-TB patients could develop drug resistance amplification to become an MDR/RR-TB case. Since the treatment success rate for new DS-TB cases (*c)* is not reported by the WHO, we used the reported treatment success rate in all new TB cases as an estimate for *c*. This is consistent with the observations that that although undiagnosed MDR/RR-TB cases (which represent a small proportion of the new cases) are less likely to achieve a favorable treatment outcome, this is counterbalanced by the observation that many patients classified as TB treatment success were not bacteriologically confirmed, which may overestimate treatment success.

Re-infection was considered possible once patients had completed treatment, with the proportion estimated from the local TB incidence using the regression equation proposed by Wang and colleagues [18]. That is, parameter *g* presented in Fig. 1 was indirectly estimated from the local TB-incidence and the other model parameters (see Additional file 1). We assumed that the proportion of MDR/RR-TB among re-infection cases was the same as that for new TB cases. Most model parameters (TB-incidence, *a*, *b*, *c*, *d, h, m* and *k*) were estimated from the Global TB Report 2016 reporting of local-level data and estimates for the year 2015, while the remaining two parameters (*e* and *f*) were estimated from the literature [2, 8, 19–23] (see Additional file 1 for details). Table 1 presents the definitions of the different parameters along with the values used for each of the WHO regions.

**Table 1:** Parameter definitions and values associated with the different WHO regions

Parameters		WHO regions						
Notation	Definition	African	American	Eastern Mediter.	European	South-East Asia	Western Pacific	Global
Inc^a^	Incidence (new cases/100,000/year)	275 (239–314)	27 (25–29)	116 (86–149)	36 (33–38)	246 (167–339)	86 (78–94)	142 (119–166)
a^a^	rate of MDR/RR-TB among new TB-cases (%)	3 (1.2-4.9)	2.9 (1.6-4.2)	4.1 (3–5.1)	16 (11–20)	2.6 (2.3-3)	5.1 (3–7.2)	3.9 (2.7-5.1)
b^b^	drug sensitivity testing coverage in new TB-cases (%)	21	29	2	44	5.1	8.8	24
c^b^	treatment success rates for new DS-TB cases (%)	81	76	91	76	79	92	83
d^b^	treatment success rates for MDR/RR-TB cases (%)	54	55	68	52	49	57	52
e^c^	treatment success rate for MDR/RR-TB treated with first-line regimen (%)	0-20	0-20	0-20	0-20	0-20	0-20	0-20
f^c^	risk of drug-resistance amplification for a DS-TB patient failing therapy (%)	10-20	10-20	10-20	10-20	10-20	10-20	10-20
h^b^	proportion of detected MDR/RR-TB cases that start on second-line regimen (%)	68.64	75.16	82.5	100	90.81	76.14	94.6
m^b^	death proportion during treatment for DS-TB and MDR/RR-TB patients (%)	5.76	6.95	1.84	7.85	3.52	2.05	3.84
k^b^	death proportion during treatment for MDR/RR-TB (%)	20.58	8.22	16.03	15.61	20.59	9.51	16.84

^a^95% Confidence Intervals as reported in the WHO TB report 2016

^b^Confidence Intervals not available

^c^Estimated from literature

### Stochastic method for generating parameter values

For the analysis by WHO region, some parameter values were associated with an uncertainty interval (Table 1). In an earlier version of this work, we also estimated uncertainty for the other parameters by using the cohort sizes reported by WHO. However, given that the cohort sizes were extremely large, the associated uncertainty intervals were very narrow, making the analysis with point estimates equivalent to that including uncertainty ranges. Therefore, we used point estimates for these parameters in the main analysis and considered broader uncertainty ranges in a supplementary analysis (see Additional file 1).

We used a stochastic Monte-Carlo method to independently generate a large number of parameter sets (1,000,000). For each run and for each parameter associated with an uncertainty interval, values were independently drawn using beta distributions with shape parameters *α* = 2 and *β* = 2, scaled and transposed in order to cover the corresponding uncertainty interval. In contrast, country-specific analysis was performed using the point estimates presented in Additional file 1: Table S2.

Similar to the region-specific analysis, parameters (TB-incidence, *a*, *b*, *c*, *d, h and m*) were estimated locally in the country-specific analysis and estimates were extracted from the tuberculosis country profiles available from WHO [24]. Where a country-specific estimate was not available and for the risk of death in MDR/RR-TB patients (*k)*, we used the value of the WHO region to which the country belongs. If three or more estimates were not accessible for one country or if the country’s incidence rate was <50 per 100,000 per year, the results of the analysis were excluded. This disease burden limitation was applied, since the epidemiology in low burden settings mostly represents imported disease and not local transmission. Therefore, we do not consider the model to be applicable to such settings.

### Observed outputs

For each geographical area, we estimated the contributions of the four different causal pathways to MDR/RR-TB at re-treatment: I) initial drug-susceptible TB with resistance amplification during treatment; II) initial MDR/RR-TB inappropriately treated as drug-susceptible TB; III) MDR/RR-TB relapse despite appropriate treatment and IV) re-infection with MDR/RR-TB. In the context of this study, drug resistance amplification is defined as acquisition of rifampicin resitance druing primary treatment. Each of these contributions was calculated by dividing the proportion of TB cases that arrive in each associated category by the total burden of MDR/RR-TB at re-treatment.

For each of the WHO regions, we observed a fifth output to assess the reliability of the model; the absolute proportion of MDR/RR-TB among re-treatment cases. We verified our model outputs against real world WHO report estimates for each of the different regions.

### Sensitivity analyses

Our analysis of the results on the seven WHO regions took into account uncertainty in the estimates of the parameters *c*, *e* and *f*, which were not directly available in the Global TB Report 2016. Thus, we observed how model outputs were impacted when treatment success rates for DS-TB (*c*) varied between 70 and 95%; probability of treatment success for MDR/RR-TB cases treated as DS-TB cases (*e*) varied between 0 and 20%; and risk of drug resistance amplification when failing first-line treatment for DS-TB (*f*) varied between 0 and 26%.

In our baseline analysis, we assumed that all MDR/RR-TB patients have the same risk of death (*k*), regardless the type of therapy received. A sensitivity analysis was performed to test this assumption by considering a modified mortality for MDR/RR-TB patients treated inappropriately. An additional sensitivity analysis was conducted to test the assumption concerning the proportion of MDR/RR-TB among re-infection cases, which was considered to be the same as for new TB cases in the baseline analysis.

Finally, in another sensitivity analysis we considered different scenarios concerning the treatment outcomes for individuals who were lost to follow-up or not evaluated.

## Results

**Fig. 2:**
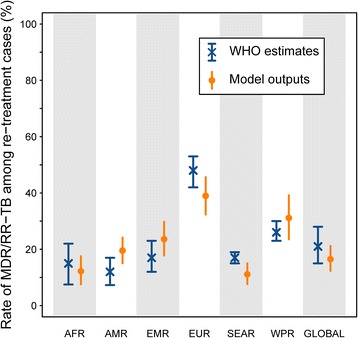
Rates of MDR/RR-TB at re-treatment by WHO region. Blue crosses show the estimates presented in the WHO Global Tuberculosis Report 2015 and vertical blue bars represent the associated 95% confidence intervals. Orange dots show the average model outputs while vertical orange bars represent the 95% central ranges obtained from the uncertainty analysis. WHO regions are designated as following: African region (AFR), American region (AMR), Eastern Mediterranean region (EMR), European region (EUR), South East Asian region (SEAR), Western Pacific region (WPR) and Global region (GLOBAL)

Figure 2 shows the proportions of MDR/RR-TB at re-treatment obtained from the model when applied to the seven WHO regions, compared to corresponding estimates provided by the WHO TB report 2016. We observed closely matching rates of MDR/RR-TB for every region.

**Fig. 3:**
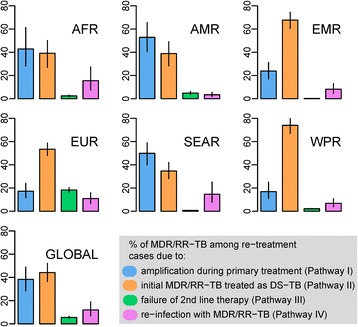
Contributions of the different causal pathways leading to MDR/RR-TB at re-treatment in the seven WHO regions. Results are expressed as percentages of the total burden of MDR/RR-TB at re-treatment. For each region, the mean values and the intervals containing 95% of the values obtained from simulation of 1,000,000 sets of parameters are presented by the bars and the lines respectively. WHO regions are designated as following: African region (AFR), American region (AMR), Eastern Mediterranean region (EMR), European region (EUR), South East Asian region (SEAR), Western Pacific region (WPR) and Global region (GLOBAL)

Figure 3 quantifies the contributions of the different causal pathways of MDR/RR-TB at re-treatment in each of the seven WHO regions. At the global level, the model suggests that the greatest number of MDR/RR-TB cases identified at re-treatment result from initial MDR/RR-TB that was inappropriately treated as DS-TB (44%, 36–52, 95% simulation interval). This was a leading pathway in every WHO region, with rates ranging from 35% (28–42) in South-East Asia to 74% (67–80) in the Western Pacific region. MDR/RR-TB at re-treatment resulting from drug resistance amplification represented 38% (28–49) of the total burden globally. Drug resistance amplification was estimated to be the leading pathway to MDR/RR-TB at re-treatment in the American, South-East Asian and African regions; accounting for 53% (40–66), 50% (40–59) and 43% (28–61) of the total burden respectively. Elsewhere, the contribution of drug resistance amplification during primary treatment ranged from 17% (11–25) (Western Pacific) to 24% (Eastern Mediterranean).

Model outputs suggested that failure of appropriate second-line regimens against MDR/RR-TB contributes little to the total burden of MDR/RR-TB at re-treatment (6% globally). The highest contribution from this pathway was found in Europe, with 18% (16–20) of the total burden of MDR/RR-TB at re-treatment. The role of re-infection was also low across all WHO regions, with an estimated global contribution of 12% (7–19); regional estimates varied from 3% (2–6) in America to 16% (8–27) in Africa.

Sensitivity analysis noted no sensitivity to parameter *e* (treatment success rate in MDR/RR-TB patients treated as DS-TB cases), whereas a lower treatment success rate for DS-TB (*c*) or a higher risk of drug resistance amplification (*f*) led to similar impacts on the results. Specifically, the main contributors to MDR/RR-TB at re-treatment would remain failure to provide appropriate anti-TB treatments and drug resistance amplification during treatment in all settings, although the respective contributions of these two pathways would be modified. The contribution of drug resistance amplification would increase, while the contribution of inappropriate diagnosis and treatment would decrease.

Our analyses including broader uncertainty ranges led to very similar results compared to the baseline analysis (see Additional file 1). The additional explorations testing our assumptions regarding both the mortality of MDR/RR-TB cases and the rate of MDR/RR-TB at re-infection demonstrate that our baseline assumptions represented at most a minimal source of bias. Finally, our analysis concerning the treatment outcomes in individuals who were lost to follow-up or not evaluated revealed that the contribution of pathway II (initial MDR/RR-TB inappropriately treated) would become more important in all WHO region if more of the unknown treatment outcomes were actually success. Detailed results of the different sensitivity analyses are presented in the Additional file 1.

**Fig. 4:**
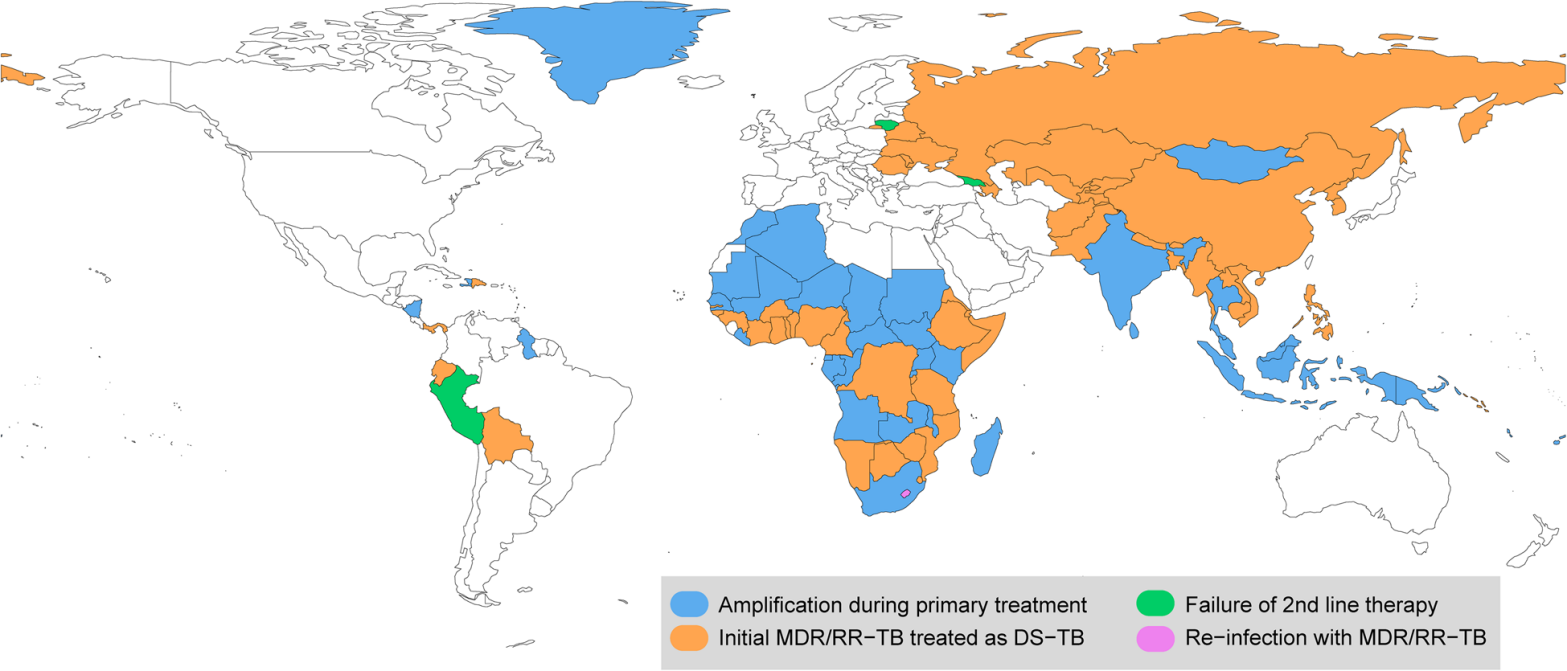
Representation of the leading pathway to MDR/RR-TB at re-treatment around the world. Only countries with TB-incidence ≥50 new cases/100,000/year and for which sufficient data was available (see the Methods section for a full description) are represented

Figure 4 presents the leading cause of MDR/RR-TB among re-treatment cases in the 105 countries with a TB-incidence ≥50 new cases/100,000/year and sufficient data for analysis. The quantitative results regarding the contribution of each of the different pathways are available in the Additional file 1. At the country-level, the leading contributor to drug resistance at re-treatment showed marked geographic variation, broadly similar to those observed in the regional analysis, but with interesting local findings. In the African region, while drug resistance amplification during primary treatment was found to be dominant in the northern part of the region, inappropriate treatment of primary MDR/RR-TB was the leading causal pathway to MDR/RR-TB at re-treatment in the countries of the Southern part of Western Africa (from Guinea Bissau to Cameroon). In the rest of the African region, we observed a relatively even division between the two main pathways leading to MDR/RR-TB at re-treatment (inappropriate therapy and drug resistance amplification). Re-infection with an MDR/RR-TB strain was the leading pathway in only one country, Lesotho, contributing 37% of cases. This cause was also common in Swaziland where it accounted for 34% of MDR/RR-TB at re-treatment. Failure of second-line regimens was the leading pathway to MDR/RR-TB at re-treatment in three countries: Lithuania (50%), Georgia (33%) and Peru (31%). In South Africa, 36% of cases were due to drug resistance amplification, while inappropriate treatment of primary MDR/RR-TB and re-infection with MDR/RR-TB contributed 23 and 33% respectively to the total burden of MDR/RR-TB at re-treatment.

## Discussion

This study is the first to use a probabilistic mathematical model to explore the different causal pathways leading to MDR/RR-TB at re-treatment and to quantify their respective contributions. By using regional and country-specific inputs, the resulting model incorporated local variations in TB control activity and disease burden. Despite these local variations, broad regional trends were observed. Most importantly, and contrary to previous dogma that emphasised the central role of drug resistance acquisition, undetected MDR/RR-TB at initial diagnosis was the most important reason for the high rates of MDR/RR-TB found among re-treatment cases in most regions. Moreover, this finding was obtained under the conservative assumption that all individuals who were lost to follow-up or not evaluated experienced treatment failure, and our sensitivity analysis demonstrates that the contribution of undetected MDR/RR-TB would increase further if some of these unknown outcomes were favourable.

Our findings therefore indicate that drug resistance amplification due to poor first-line treatment adherence is not the predominant pathway to MDR/RR-TB at re-treatment. Instead, the greatest number of MDR/RR-TB cases result from transmission of MDR/RR-TB strains and it is the lack of appropriate MDR/RR-TB identification at initial presentation that underlies the high rates of MDR/RR-TB at re-presentation. These observations emphasize the importance of universal MDR/RR-TB screening followed by rapid initiation of appropriate treatment. They therefore highlight the potential impact that novel rapid diagnostic tests that include rifampicin sensitivity testing such as Xpert MTB/RIF® could have on the MDR-TB epidemic. Our findings concur with the outcomes of recent studies demonstrating that primary transmission contributes more substantially to the MDR/RR-TB burden than drug resistance acquisition or amplification during treatment [17, 25]. Interestingly, the model also suggests that failure of second-line regimens may make a significant contribution to the burden of MDR/RR-TB at re-treatment, especially in settings such as Eastern Europe where MDR vigilance is high and patients are able to access MDR/RR-TB treatment. Our analysis has crucial implications for TB control as it highlights regional variation regarding the causes of MDR/RR-TB at re-treatment, indicating that targeted programmatic strategies may be more effective than elaborating a single global plan. In all regions and particularly in the Western Pacific and the Eastern Mediterranean, , the introduction of universal drug resistance screening of newly diagnosed TB cases seems critical. Universal drug resistance screening would reduce the burden of MDR/RR-TB at re-treatment and limit on-going MDR/RR-TB transmission within the community, as well as directly benefiting patients who would otherwise have been inappropriately managed with first-line treatment during their initial disease episode.

Access to appropriate therapy is crucial as DS-TB cases that are not properly treated may experience drug resistance amplification. In the Americas, South-East Asia and Africa, the greatest contribution of MDR/RR-TB identified at re-treatment resulted from drug resistance amplification during first-line treatment of DS-TB. Amplification is driven by high treatment failure rates on first-line therapy, which indicates that improved treatment adherence should be a major public health priority in these settings. The DOTS strategy has demonstrated ability to improve treatment outcomes and therefore, meticulous scale-up should reduce the MDR/RR-TB burden in these regions [26–29]. This requires strong political commitment together with substantial and sustainable financing, especially in low and middle income countries.

Re-infection with MDR/RR-TB was found to play an important role in Lesotho, Swaziland and South Africa, which were the only countries where it contributed more than one third to the total burden of MDR/RR-TB at re-treatment. This may be explained by the exceptional infection pressure that exists in such settings with a TB-incidence ranging between 565 and 834 cases/100,000 people in 2015 [2]. Accordingly, in settings with very high infection pressure, as occur in particular disease “hot-spots”, a more comprehensive response is required to limit TB transmission and case numbers of both DS-TB and MDR/RR-TB. In these three countries of Southern Africa, such an approach would include better control of the severe HIV epidemic, enhanced active case finding strategies, and creative interventions to reduce TB transmission within communities [30]. In South Africa, transmission of MDR/RR-TB caused around 64% of MDR/RR -TB cases diagnosed at re-treatment, which is supported by molecular epidemiology studies indicating substantial clonal spread of multiple MDR-TB strains [11]. In addition to a strong emphasis on improved treatment adherence, consideration should be given to additional efforts that may reduce TB transmission within disease “hot spots” [31, 32].

Despite the relative simplicity of our model, uncertainty analysis demonstrated robust conclusions across plausible parameter ranges. We are also reassured by the comparison between model outputs and independently calculated WHO estimates of the total rate of MDR/RR-TB at re-treatment, which demonstrates very close approximation at both the global and region-specific level. Moreover, the model was most sensitive to variation in parameters for which programmatic (treatment success rate in new DS-TB cases, *c*) or evidence-based (risk of drug resistance amplification during treatment, *f*) data were available and of reasonable quality, whereas it was insensitive to variation in the most uncertain parameter (treatment success rate for MDR/RR -TB treated as DS-TB, *e*). In particular, the sensitivity analyses demonstrated that a lower treatment success rate for DS-TB and a higher risk of drug resistance amplification would both contribute to a higher contribution of drug resistance amplification to the burden of MDR/RR-TB at re-treatment.

Model limitations include the fact that only two phenotypes of TB were considered, DS and MDR/RR-TB. Other profiles such as mono- or poly-resistant, or additional resistance beyond MDR-TB were reclassified into these two categories, since the available WHO data do not provide additional sub-classification. Further investigations could be conducted in settings where more detailed drug resistance profile data are available. However, our sensitivity analyses indicate that our general conclusions are maintained in settings with high prevalence of mono- or poly-resistant TB. In such settings, treatment success rates for these strains are expected to be lower, while the risk of drug resistance amplification leading to MDR/RR-TB would increase. Our sensitivity analyses indicate that in these settings, the contribution of drug resistance amplification during primary treatment would increase, while the contribution of inappropriate diagnosis and treatment would diminish. Another limitation is linked to the uncertainty around some parameter estimates, in particular those that were not directly available from the WHO. As discussed above, although our results were sensitive to some model parameters, we do not believe this would jeopardise our general findings. Our model does not take into account nosocomial transmission of MDR/RR-TB. While we acknowledge this as a possible cause of MDR/RR-TB presentation at re-treatment [33, 34], insufficient data were available to inform its isolated contribution at the local level. Nevertheless, we can anticipate that our model may underestimate the contribution of reinfection in settings where nosocomial transmission of MDR/RR-TB is significant. Future works could investigate this issue more specifically and distinguish the contribution of nosocomial transmission from that of general reinfection with MDR/RR-TB strains.

The resolution of our analysis was restricted to the national level and we were unable to consider sub-national heterogeneity. Moreover, our regional-level estimates were based on common analyses of aggregate data due to missing country-level data and as countries were excluded from the analysis if TB incidence was <50 cases/100,000/year. This may not lead to the same estimates as aggregating the results of separate analyses of the different countries as the model that we use is non-linear. National TB programs need to consider particular settings within the country, since transmission dynamics may be altered within “hot-spot” areas, while cultural issues and specific service delivery challenges also require consideration [35, 36]. In future, our model could be adapted to guide local policies, for example through an online tool usable by policy makers who could input parameters from local programmatic data. Such a tool could be modified in real-time as new data become available to improve and update parameter estimates. Indeed it is important to note that our estimates correspond to the situation in 2015 and that the fractions attributable to each pathway are likely to vary over time.

## Conclusions

Our findings highlight the need for contextualised solutions to limit the impact and spread of MDR/RR-TB. Although more effective MDR/RR-TB treatment is a universal need and certain common factors should be addressed, a better understanding of the local causal pathways could assist better targeted public health responses. Importantly, our findings suggest that simply “turning off the tap” through improved programmatic management of drug-susceptible TB will be insufficient to contain the spread of drug-resistant TB.

## Additional file

Additional file 1: Additional details on methods and sensitivity analyses. (DOCX 609 kb)

## Abbreviations

DST: Drug susceptibility testing
DS-TB: Drug-susceptible tuberculosis
MDR/RR-TB: Multidrug-resistant or rifampicin-resistant tuberculosis
TB: Tuberculosis
WHO: World Health Organization
XDR-TB: Extensively drug-resistant tuberculosis
