# Supplementary material for: High rates of multidrug-resistant and rifampicin-resistant tuberculosis among re-treatment cases: where do they come from?
Source: BMC Infect Dis. 2017 Jan 6;17:36. doi: 10.1186/s12879-016-2171-1 (PMC5217596; doi:10.1186/s12879-016-2171-1)
Supplement: Additional file 1: — Additional details on methods and sensitivity analyses. (DOCX 609 kb) [file 12879_2016_2171_MOESM1_ESM.docx]

**High Rates of Multidrug-Resistant and Rifampicin-Resistant Tuberculosis among Re-Treatment Cases: Where do they Come from?**

*Additional file*

Romain Ragonnet, James M. Trauer, Justin T. Denholm, Ben J. Marais, Emma S. McBryde

**Contents**

1 Method used for estimating the risk of re-infection p. 1

2 Use of increased uncertainty around parameter values p. 1

3 Results of the sensitivity analyses p. 3

4 Parameter values and results of analyses by region and by country p. 7

5 Appendix references p. 13

**1 Method used for estimating the risk of re-infection**

The proportion of recurrent cases that are due to re-infection is calculated from the regression equation proposed in Wang et al.[^1^](#_ENREF_1) taking local incidence as predictive variable:

$$Rp=-29.7+36.8\times log(Inc)$$

$Rp$: Re-infection proportion $Inc$: Local TB-incidence

In our model, the proportion$Rp$ is expressed as:

$$Rp=\frac{\left[ \left( 1-a \right).c+a.\left( 1-b \right).e+a.b.d \right]\times g}{\left[ \left( 1-a \right).c+a.\left( 1-b \right).e+a.b.d \right]\times g+\left( 1-a \right).\left( 1-c-m \right)+a.\left( 1-b \right).\left( 1-e-k \right)+a.b.(1-d-k)}$$

Then an estimate for the parameter $g$ is given by:

$$g=\frac{\left( 1-a \right).\left( 1-c-m \right)+a.\left( 1-b \right).\left( 1-e-k \right)+a.b.(1-d-k)}{\left( 1-a \right).c+a.\left( 1-b \right).e+a.b.d}\times\frac{Rp}{1-Rp}$$

In the analysis involving the WHO regions, uncertainty is included at two different levels:

- The level of incidence is drawn from a beta distribution ($\alpha=\beta=2$) on the 95% confidence intervals presented in the WHO TB report 2016.[^2^](#_ENREF_2)
- The regression multiplier is generated by a beta distribution ($\alpha=\beta=2$) on the interval [29.4, 44.2] corresponding to the baseline value 36.8 ±20%.

**2 Use of increased uncertainty around the parameter values**

In this analysis, we add uncertainty to the parameters that were only considered as point estimates in the baseline analysis (*b*, *c*, *d*, *h*, *m*, and *k*). To this end, the parameter values are generated from beta distributions that are defined as follows.

When $x$ designates the point estimate for the parameter value and $N$ an integer, we use the shape parameters $\alpha=Nx$ and $\beta=N(1-x)$. This approach is equivalent to what could be used to estimate the distribution of a success probability where the observed value is $x$ during an experiment that includes $N$ repetitions.

Table S1 presents the 95% confidence intervals obtained for the parameter values when using this approach with $N=1,000$, $N=500$ and $N=200$. Such values are much lower than the cohort sizes reported by the WHO for the different parameters in the different regions, suggesting that this analysis may overestimate the uncertainty associated with the data.

**Table S1. 95% confidence intervals used around the parameter estimates when using increased uncertainty.**

| **WHO Region** | **N** | **b**  **(%, CI^ii^)** | **c**  **(%, CI^ii^)** | **d**  **(%, CI^ii^)** | **h**  **(%, CI^ii^)** | **m**  **(%, CI^ii^)** | **k**  **(%, CI^ii^)** |
| --- | --- | --- | --- | --- | --- | --- | --- |
| **AFR** | -  1,000  500  200 | 21 (18.54-23.59) (17.53-24.71) (15.63-26.87) | 81  (78.53-83.36)  (77.43-84.3)  (75.29-86.11) | 54 (50.9-57.07) (49.62-58.34) (47.04-60.78) | 68.64 (65.74-71.48) (64.53-72.62) (62.04-74.9) | 5.76 (4.41-7.28) (3.9-7.97) (2.99-9.39) | 20.58 (18.15-23.16) (17.14-24.24) (15.27-26.51) |
| **AMR** | -  1,000  500  200 | 29 (26.22-31.84) (25.09-33.07) (22.94-35.5) | 76 (73.3-78.59) (72.15-79.64) (69.84-81.66) | 55 (51.94-58.09) (50.64-59.34) (48.09-61.85) | 75.16 (72.43-77.8) (71.3-78.86) (68.94-80.85) | 6.95 (5.46-8.59) (4.9-9.32) (3.86-10.82) | 8.22 (6.61-10) (5.98-10.78) (4.84-12.39) |
| **EMR** | -  1,000  500  200 | 2 (1.23-2.96) (0.96-3.39) (0.55-4.34) | 91 (89.14-92.7) (88.38-93.36) (86.65-94.56) | 68 (65.06-70.85) (63.84-72.01) (61.39-74.26) | 82.5 (80.08-84.8) (79.03-85.71) (76.97-87.44) | 1.84 (1.1-2.76) (0.85-3.18) (0.47-4.11) | 16.03 (13.83-18.37) (12.95-19.37) (11.32-21.42) |
| **EUR** | -  1,000  500  200 | 44 (40.94-47.07) (39.66-48.37) (37.24-50.9) | 76 (73.3-78.6) (72.14-79.62) (69.86-81.62) | 52 (48.91-55.07) (47.62-56.33) (45.08-58.9) | 100 (100-100) (100-100) (100-100) | 7.85 (6.27-9.58) (5.66-10.37) (4.55-11.91) | 15.61 (13.43-17.92) (12.58-18.91) (10.96-20.93) |
| **SEAR** | -  1,000  500  200 | 5.1 (3.82-6.55) (3.35-7.18) (2.51-8.55) | 79 (76.43-81.46) (75.33-82.45) (73.1-84.38) | 49 (45.9-52.08) (44.64-53.39) (42.11-55.89) | 90.81 (88.95-92.51) (88.13-93.18) (86.4-94.4) | 3.52 (2.47-4.74) (2.09-5.29) (1.44-6.46) | 20.59 (18.14-23.17) (17.16-24.23) (15.3-26.45) |
| **WPR** | -  1,000  500  200 | 8.8 (7.13-10.64) (6.47-11.42) (5.29-13.1) | 92 (90.24-93.6) (89.47-94.2) (87.86-95.32) | 57 (53.91-60.06) (52.65-61.3) (50.14-63.76) | 76.14 (73.45-78.74) (72.31-79.8) (70.06-81.78) | 2.05 (1.27-3.01) (1-3.47) (0.57-4.42) | 9.51 (7.78-11.39) (7.09-12.22) (5.86-13.97) |
| **GLOBAL** | -  1,000  500  200 | 24 (21.4-26.69) (20.35-27.84) (18.41-30.13) | 83 (80.62-85.25) (79.58-86.16) (77.47-87.86) | 52 (48.9-55.08) (47.62-56.37) (45.1-58.89) | 94.6 (93.13-95.91) (92.47-96.41) (91.1-97.27) | 3.84 (2.74-5.12) (2.33-5.7) (1.64-6.9) | 16.84 (14.58-19.21) (13.7-20.26) (12-22.34) |

Figure S1 represents a comparison of the results obtained with and without inclusion of increased uncertainty. The central estimates obtained from the different approaches are the same. The black bars represent the uncertainty obtained in the baseline analysis while the colored bars represent the uncertainty obtained when using the approach described above for different values of *N* (1000, 500 and 200). We note that the central estimates obtained from the different methods are the same and the amplitude of the uncertainty ranges around the model outputs are not significantly impacted when using increased uncertainty for the model parameters.


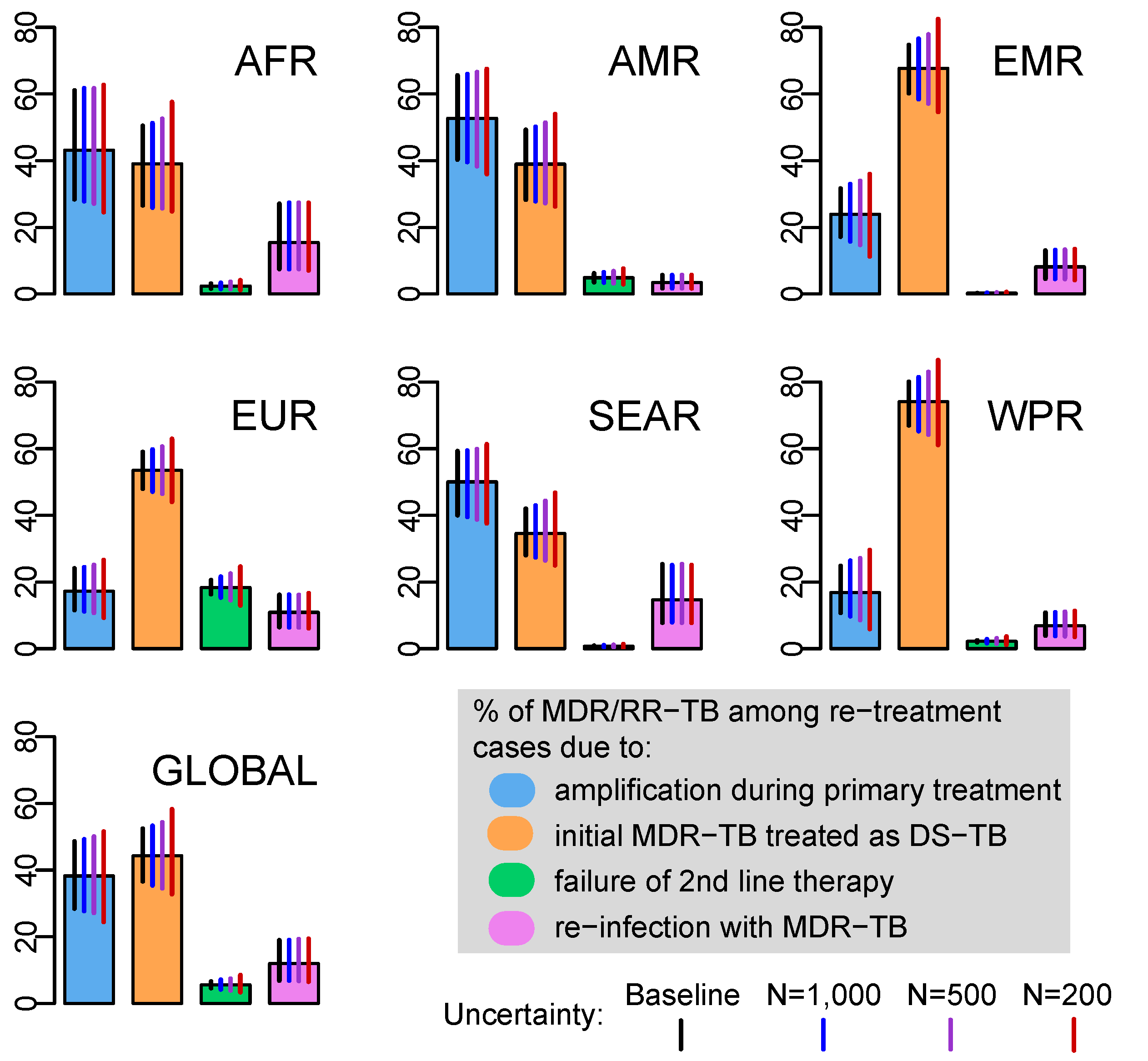


**Figure S1. Comparison of the results obtained with and without inclusion of increased uncertainty.**

The bars represent the uncertainty obtained in model outputs when using different approaches to include uncertainty in model parameters.

**3 Results of the sensitivity analyses**

Figure S2 presents the results of the sensitivity analysis performed for the parameters *c*, *e* and *f*. In every region, considering higher treatment success rates for DS-TB (*c*) results in fewer cases of drug resistance amplification (DRA), which is intuitive given that patients only amplify resistance if treatment fails under our model. Consequently, a reduction in DRA is compensated for by an increase in the contributions of the two other causes involving patients that were initially MDR/RR-TB cases and vice versa. In Europe – where DST coverage is high – we observe that this increase is equally distributed between the two categories “initial MDR/RR-TB treated with a failing first line regimen” and “initial MDR/RR-TB treated with a failing second line regimen”. By contrast, in the other regions – where DST coverage is either low or very low – only the contribution of initial MDR/RR-TB cases treated with a failing first line regimen increases, as fewer patients received second line regimen.

As parameter *f* increases (the risk of DRA among DS-TB patients in whom treatment fails), the contribution of DRA to the burden of MDR/RR-TB at re-treatment unsurprisingly increases in every region. As described for parameter *c*, changes in parameter *f* lead to compensatory changes in the two other contributing factors. As *f* increases, there is a combined decrease distributed between the two categories “initial MDR-TB treated with a failing first line regimen” and “initial MDR/RR-TB treated with a failing second line regimen”. This distribution is evenly shared for Europe, while the decrease only affects the contribution of initial MDR/RR-TB cases treated with a failing first line regimen for the other regions, for the same reasons outlined above for parameter *c*.

**
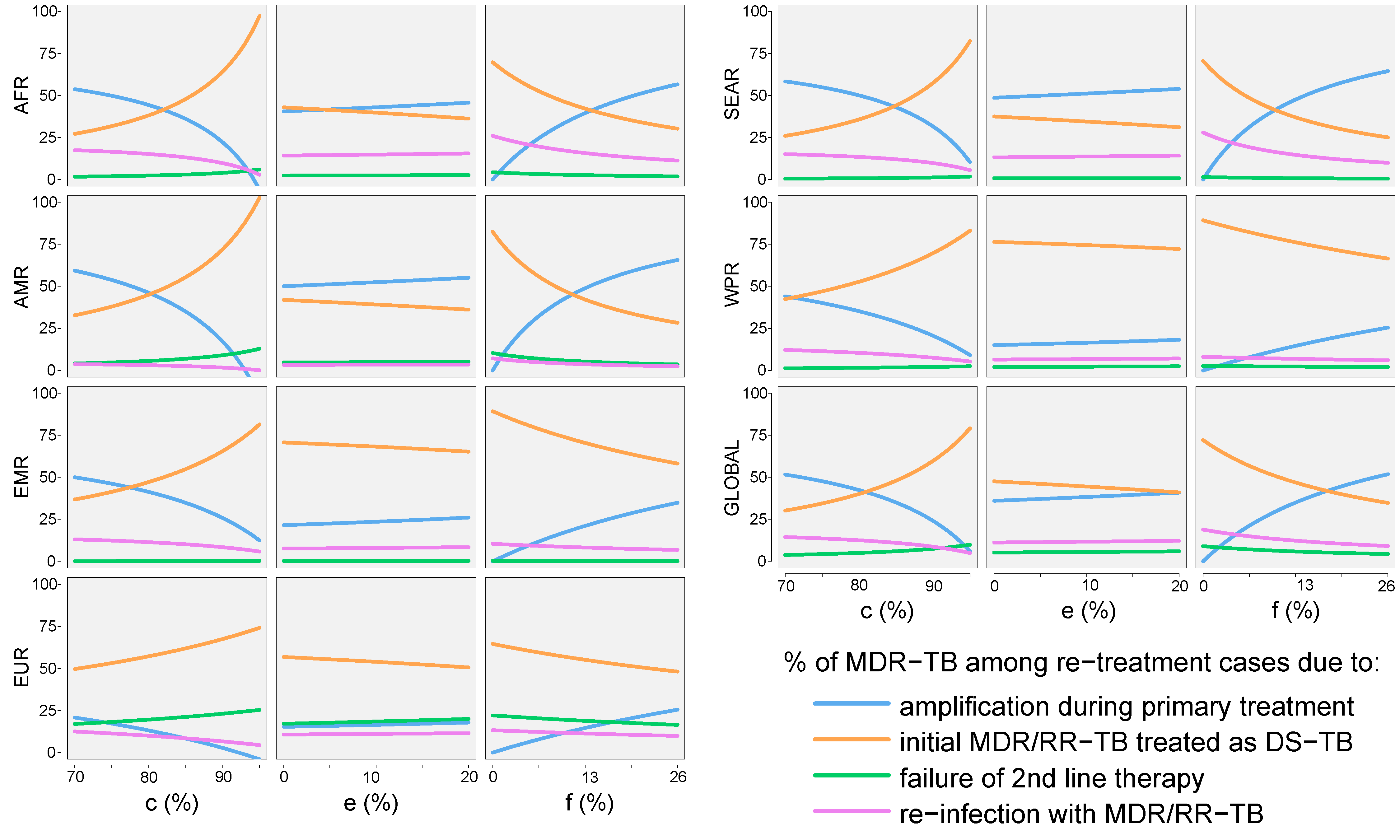
**

**Figure S2. Results of the sensitivity analysis on the parameters *c*, *e* and *f*.**

Variability in the contributions of the different pathways to MDR/RR-TB at re-treatment when considering different values for the treatment success rate against DS-TB (*c*); for the treatment success rate against MDR/RR-TB treated with 1^st^ line regimen (*e*); and for the risk of drug resistance amplification among DS-TB patients in whom treatment failed (*f*). Results are presented as percentages of the total burden of MDR/RR-TB at re-treatment. WHO regions are designated as following: African region (AFR), American region (AMR), Eastern Mediterranean region (EMR), European region (EUR), South East Asian region (SEAR), Western Pacific region (WPR) and Global region (GLOBAL).

Two additional sen**s**itivity analyses described below were performed in order to test alternate assumptions in our model.

First, in the baseline analysis, we assumed that all MDR/RR-TB patients had the same risk of death (parameter $k$), regardless the type of therapy received. However, one may consider that MDR/RR-TB patients receiving first-line regimens are at higher risk of death than appropriately treated patients given that their infection is more likely to persist. On the other hand, patients who are treated with second-line regimens are exposed to a higher level of toxicity induced by the drugs, which may therefore increase their risk of death. The fact that these two phenomena represent compensative effects for the risk of death demonstrates that it was reasonable to assume a same mortality for all MDR/RR-TB patients. Nevertheless, in this sensitivity analysis, we explore the effect of considering alternate assumptions regarding the mortality in MDR/RR-TB patients. Namely, we varied the relative risk of death for MDR/RR-TB patients receiving first-line regimen between 0.5 and 1.5. Figure S3 represents the corresponding variations in the model outputs.

**
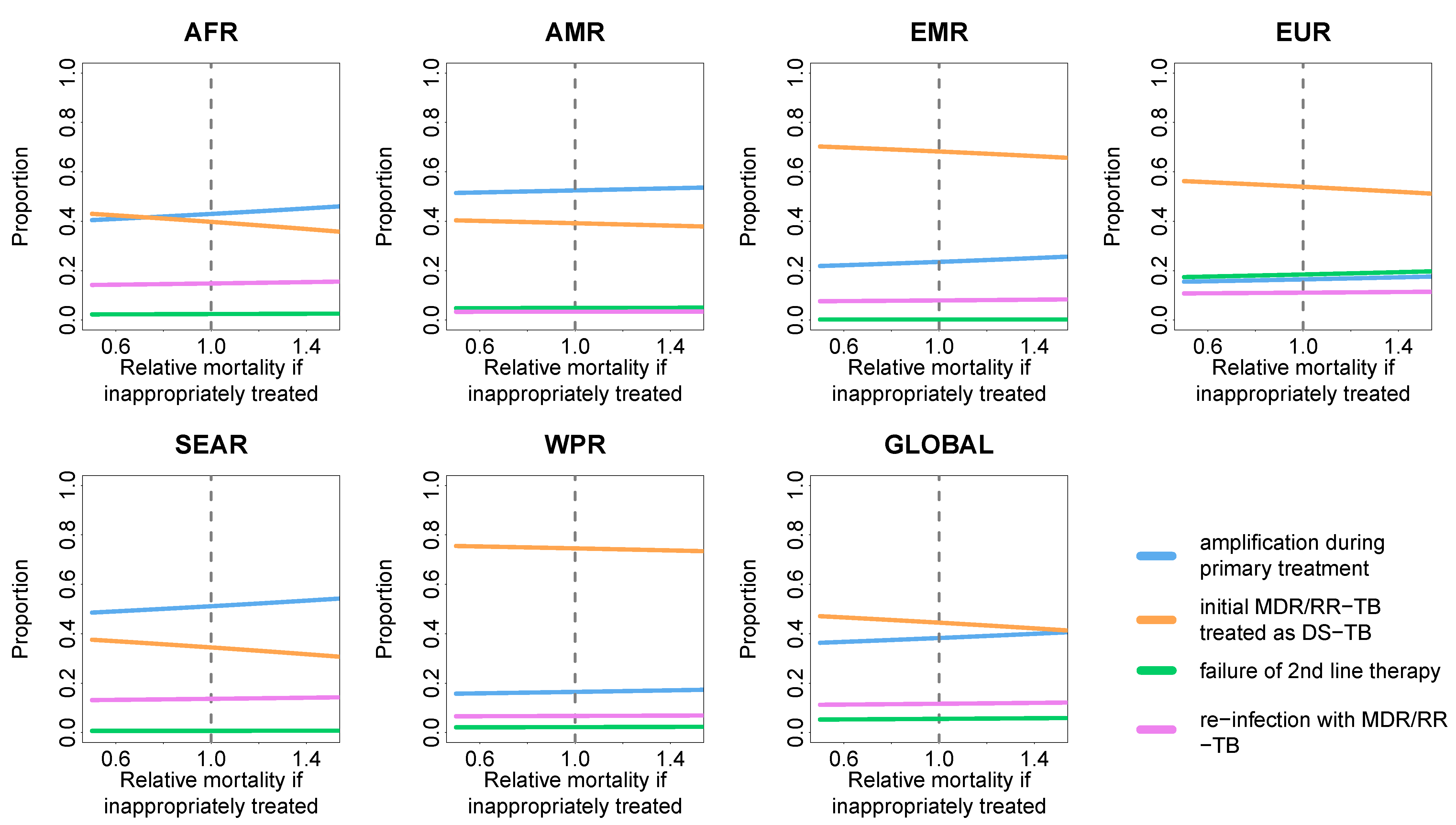
**

**Figure S3. Results of the sensitivity analysis for the relative mortality in MDR/RR-TB patient on first-line regimen.**

The vertical dashed line represents the baseline scenario with a same risk of death in all MDR/RR-TB patients.

We note that considering alternate scenarios concerning the risk of death in inappropriately treated MDR/RR-TB patients does not lead to substantial changes in the findings and therefore conclude that our initial assumption does not constitute a source of bias for our conclusions.

The second assumption that we tested as a sensitivity analysis concerns the risk of contracting MDR/RR-TB during a re-infection episode. In the initial analysis, we assumed that this risk was equal to the rate of MDR/RR-TB in new cases (parameter *a*) as we are not aware of any reason why this assumption should be invalid. Here we explore alternate scenarios where the relative risk of MDR/RR-TB in re-infection cases compared to the one in new cases varies between 0.5 and 1.5. Figure S4 represents the results of this exploration.

**
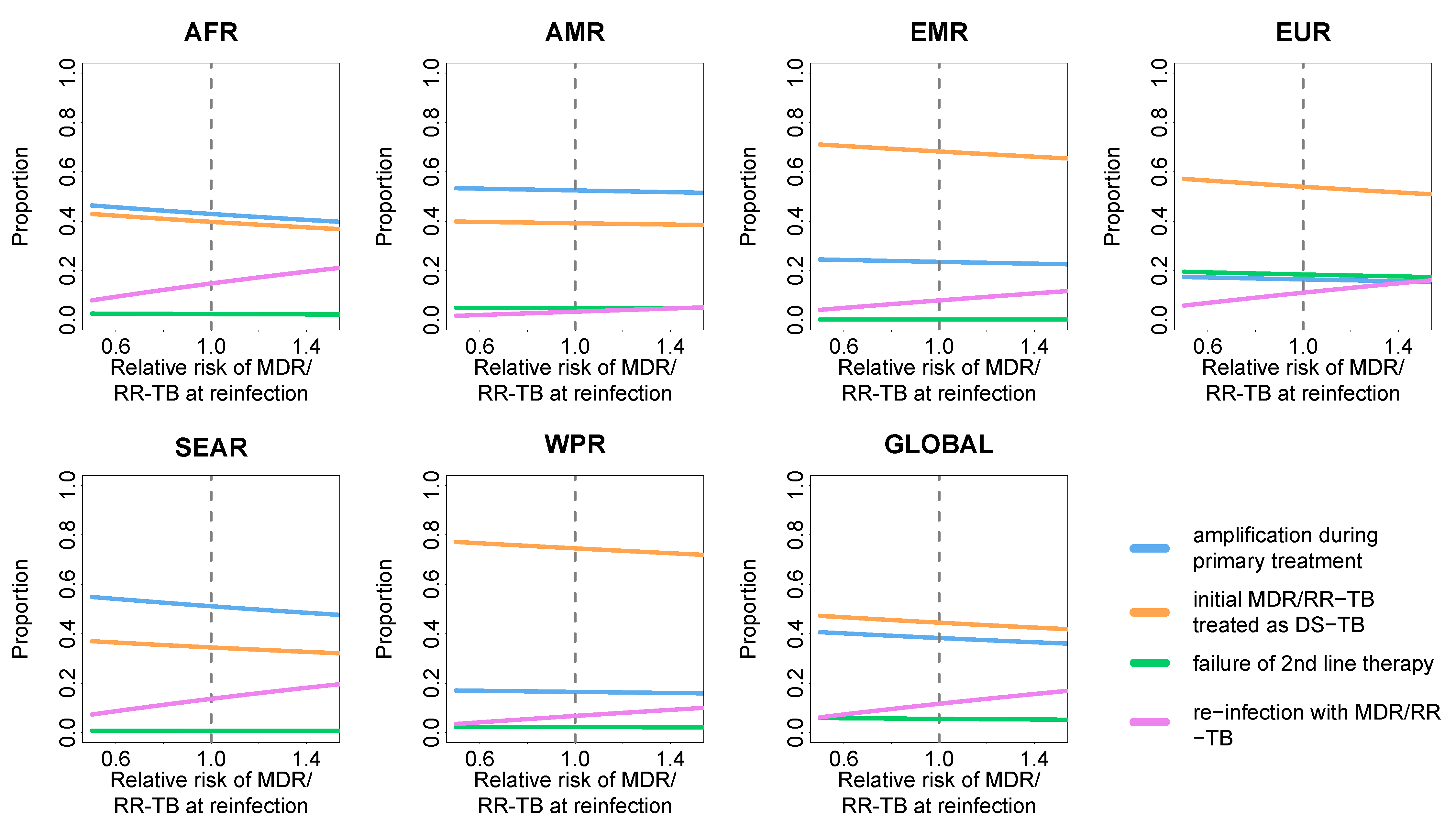
**

**Figure S4.** **Results of the sensitivity analysis for the relative risk of MDR/RR-TB in re-infection patients.** The vertical dashed line represents the baseline scenario where the risk of MDR/RR-TB in new and re-infection cases was the same.

We observe that the contribution of re-infection to the burden of MDR-TB at re-treatment only increases slightly when we consider an augmented risk of MDR-TB for re-infection cases compared to new cases. Indeed, the greatest change induced by the consideration of an alternate assumption would be for the African region but its amplitude would be very limited, even when considering a relative risk of 1.5. In this case, the contribution of re-infection would reach 20% while it was estimated at 16% in the initial analysis. At the Global level, the contribution of re-infection would only increase by 5% compared to the baseline estimate if we assumed a relative risk of MDR-TB at re-infection of 1.5 (18% vs. 12%).

We can conclude that our findings would not be jeopardized by alternate assumptions concerning the risk of MDR-TB at re-infection.

In another sensitivity analysis, we investigated alternate assumptions concerning the treatment outcome of individuals classified as lost to follow-up or not evaluated. While these individuals were assumed to experience treatment failure in our baseline analysis, we now consider different assumptions that allow for treatment success for a proportion of them (up to 50%). Figure S5 represents the results of this analysis.

This analysis highlights the fact that misdiagnosis and inappropriate treatment of primary MDR/RR-TB cases would become even more important contributor to the burden of MDR/RR-TB at re-treatment if we considered positive treatment outcomes for individuals who are lost to follow-up or not evaluated. In the African region for example, inappropriate treatment of primary MDR/RR-TB would become the leading cause of MDR/RR-TB at re-treatment if more than 10% of unknown treatment outcomes were positive.


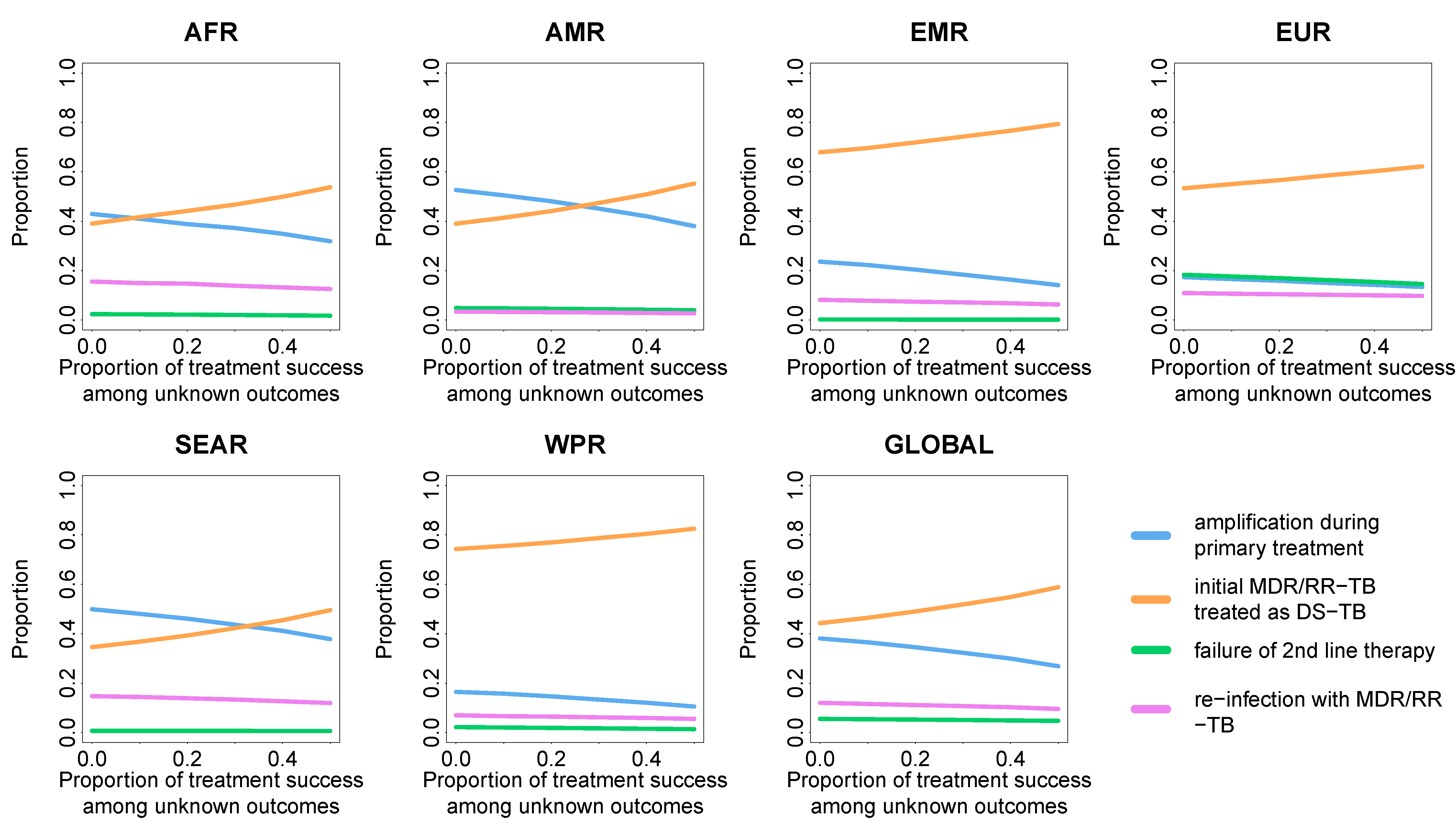


**Figure S5.** **Results of the sensitivity analysis on the treatment outcomes experienced by individuals lost to follow-up or not evaluated.**

**4 Parameter values and results of analyses by region and by country**

Table S1 presents the parameter values used in our analyses as well as the results regarding the contributions of the different mechanisms leading to MDR-TB at re-treatment. Local estimates come from the Global TB report 2016 of the WHO. The background colors that are used in the columns of Table S1 correspond to the color code that we use in the figures 3 and 4 for designating the different causes of MDR-TB at re-treatment.

**Table S2. Parameter values and results related to the different WHO regions and countries.**

|  | **Parameters** | | | | | | | | **% or MDR/RR-TB at re-treatment due to** | | | |
| --- | --- | --- | --- | --- | --- | --- | --- | --- | --- | --- | --- | --- |
| **WHO region** | **Incidence**  **(cases/100,000/y)** | **a**  **(%, CI^i^)** | **b**  **(%, CI^ii^)** | **c**  **(%, CI^ii^)** | **d**  **(%, CI^ii^)** | **h**  **(%, CI^ii^)** | **m**  **(%, CI^ii^)** | **k**  **(%, CI^ii^)** | **Amplification during treatment** | **Inappropriate regimen** | **Appropriate regimen failing** | **Reinfection with MDR/RR-TB strain** |
| AFR | 275 (239-314) | 3 (1.2-4.9) | 21 | 81 | 54 | 68.64 | 5.76 | 20.58 | 43 (28-61) | 39 (27-50) | 2 (2-3) | 16 (8-27) |
| AMR | 27 (25-29) | 2.9 (1.6-4.2) | 29 | 76 | 55 | 75.16 | 6.95 | 8.22 | 53 (40-66) | 39 (28-49) | 5 (4-6) | 3 (2-6) |
| EMR | 116 (86-149) | 4.1 (3-5.1) | 2 | 91 | 68 | 82.5 | 1.84 | 16.03 | 24 (17-31) | 68 (60-74) | 0 (0-0) | 8 (5-13) |
| EUR | 36 (33-38) | 16 (11-20) | 44 | 76 | 52 | 100 | 7.85 | 15.61 | 17 (12-24) | 53 (48-59) | 18 (16-20) | 11 (6-16) |
| SEAR | 246 (167-339) | 2.6 (2.3-3) | 5.1 | 79 | 49 | 90.81 | 3.52 | 20.59 | 50 (40-59) | 35 (28-42) | 1 (1-1) | 15 (8-25) |
| WPR | 86 (78-94) | 5.1 (3-7.2) | 8.8 | 92 | 57 | 76.14 | 2.05 | 9.51 | 17 (11-25) | 74 (67-80) | 2 (2-2) | 7 (4-11) |
| GLOBAL | 142 (119-166) | 3.9 (2.7-5.1) | 24 | 83 | 52 | 94.6 | 3.84 | 16.84 | 38 (28-49) | 44 (36-52) | 6 (5-7) | 12 (7-19) |
|  |  |  |  |  |  |  |  |  |  |  |  |  |
| **Country** | **Incidence**  **(cases/100,000/y)** | **a (%)** | **b (%)** | **c (%)** | **d (%)** | **h (%)** | **m (%)** | **k (%)** | **Amplification during treatment** | **Inappropriate regimen** | **Appropriate regimen failing** | **Reinfection with MDR/RR-TB strain** |
| Afghanistan | 189 | 3.90 | 0.00 | 87.00 | 63.00 | 100.00 | 1.28 | - | 32 | 55 | 0 | 12 |
| Algeria | 75 | 1.40 | 2.00 | 88.00 | - | 100.00 | 1.70 | - | 59 | 37 | 0 | 4 |
| Angola | 370 | 2.80 | - | 34.00 | 74.00 | 100.00 | 2.65 | - | 79 | 13 | 0 | 8 |
| Azerbaijan | 69 | 13.00 | 32.00 | 83.00 | 59.00 | 70.62 | 3.39 | - | 15 | 65 | 6 | 14 |
| Bangladesh | 225 | 1.60 | 5.00 | 93.00 | 75.00 | 92.24 | 3.79 | - | 29 | 65 | 0 | 5 |
| Belarus | 55 | 37.00 | 69.00 | 88.00 | 54.00 | 100.00 | 5.28 | - | 3 | 41 | 37 | 19 |
| Benin | 60 | 1.20 | 8.00 | 89.00 | 93.00 | 82.61 | 5.84 | - | 44 | 54 | 0 | 2 |
| Bhutan | 155 | 2.60 | 53.00 | 90.00 | 92.00 | 100.00 | 4.13 | - | 42 | 49 | 0 | 9 |
| Bolivia | 117 | 3.00 | 6.00 | 83.00 | 61.00 | 51.90 | 5.32 | - | 38 | 53 | 1 | 8 |
| Botswana | 356 | 3.60 | <1 | 77.00 | 71.00 | 100.00 | 8.73 | - | 37 | 44 | 0 | 19 |
| Brunei Darussalam | 58 | 0.00 | 79.00 | 65.00 | 0.00 | - | 7.07 | - | 100 | 0 | 0 | 0 |
| Burkina Faso | 52 | 2.80 | 2.00 | 81.00 | 62.00 | 66.67 | 9.53 | - | 40 | 55 | 0 | 5 |
| Burundi | 122 | 3.20 | 9.00 | 91.00 | 89.00 | 93.18 | 5.77 | - | 16 | 79 | 0 | 5 |
| Cambodia | 380 | 1.80 | <1 | 93.00 | 75.00 | 97.40 | 1.87 | - | 31 | 60 | 0 | 9 |
| Cameroon | 212 | 3.20 | <1 | 84.00 | 92.00 | 100.00 | 7.39 | - | 29 | 61 | 0 | 10 |
| Central African Republic | 391 | 0.40 | <1 | 70.00 | 81.00 | 61.29 | 5.90 | - | 88 | 7 | 0 | 5 |
| Chad | 152 | 2.80 | 1.00 | 68.00 | - | 100.00 | 3.38 | - | 60 | 28 | 0 | 12 |
| China | 67 | 6.60 | 8.00 | 94.00 | 55.00 | 58.90 | 1.01 | - | 11 | 81 | 2 | 6 |
| China Macao | 72 | 2.50 | 75.00 | 86.00 | 88.00 | 45.45 | 6.75 | - | 42 | 52 | 1 | 5 |
| Congo | 379 | 3.20 | - | 69.00 | - | 31.71 | 1.56 | - | 52 | 25 | 1 | 22 |
| Cote d’Ivoire | 159 | 3.10 | - | 79.00 | 85.00 | 84.38 | 10.41 | - | 40 | 50 | 0 | 10 |
| Democratic People’s Republic of Korea | 561 | 2.20 | 0.00 | 91.00 | 84.00 | 59.81 | 3.11 | - | 30 | 56 | 0 | 14 |
| Congo | 324 | 3.20 | 2.00 | 89.00 | 63.00 | 82.77 | 4.16 | - | 27 | 60 | 0 | 13 |
| Djibouti | 378 | 4.30 | 5.00 | 81.00 | - | 83.53 | 2.18 | - | 34 | 43 | 0 | 22 |
| Dominican Republic | 60 | 3.00 | 7.00 | 83.00 | 73.00 | 100.00 | 4.84 | - | 41 | 53 | 1 | 5 |
| Ecuador | 52 | 7.30 | 20.00 | 77.00 | 45.00 | 21.26 | 4.95 | - | 27 | 62 | 2 | 9 |
| Equatorial Guinea | 172 | 2.80 | 11.00 | 58.00 | - | 66.67 | 3.27 | - | 65 | 21 | 1 | 14 |
| Eritrea | 65 | 2.80 | - | 91.00 | 83.00 | 100.00 | 4.65 | - | 27 | 69 | 0 | 4 |
| Ethiopia | 192 | 2.70 | 9.00 | 89.00 | 68.00 | 100.00 | 3.14 | - | 36 | 54 | 1 | 9 |
| Fiji | 51 | 0.00 | 6.00 | 87.00 | - | - | 8.25 | - | 100 | 0 | 0 | 0 |
| Gabon | 465 | 3.20 | 1.00 | 58.00 | - | 26.67 | 1.49 | - | 59 | 22 | 0 | 18 |
| Gambia | 174 | 2.80 | 0.00 | 88.00 | - | 0.00 | 6.10 | - | 28 | 64 | 0 | 8 |
| Georgia | 99 | 12.00 | 65.00 | 83.00 | 43.00 | 100.00 | 3.25 | - | 18 | 32 | 33 | 17 |
| Ghana | 160 | 2.80 | 5.00 | 85.00 | 69.00 | 83.33 | 10.26 | - | 25 | 68 | 0 | 7 |
| Greenland | 164 | 2.30 | 0.00 | 68.00 | - | - | 3.03 | - | 62 | 27 | 0 | 11 |
| Guam | 51 | 0.00 | 57.00 | 89.00 | - | 50.00 | 3.57 | - | 100 | 0 | 0 | 0 |
| Guinea | 177 | 2.80 | 1.00 | 83.00 | 58.00 | 27.64 | 5.40 | - | 42 | 48 | 0 | 10 |
| Guinea-Bissau | 373 | 2.80 | 10.00 | 81.00 | 40.00 | 88.24 | 8.42 | - | 38 | 44 | 2 | 16 |
| Guyana | 93 | 3.00 | 5.00 | 69.00 | - | 100.00 | 9.72 | - | 52 | 39 | 1 | 9 |
| Haiti | 194 | 3.00 | 7.00 | 78.00 | 83.00 | 88.24 | 4.73 | - | 46 | 42 | 0 | 12 |
| India | 217 | 2.50 | 6.00 | 74.00 | 46.00 | 93.39 | 3.52 | - | 57 | 29 | 1 | 13 |
| Indonesia | 395 | 2.80 | 0.50 | 84.00 | 51.00 | 71.15 | 2.29 | - | 42 | 41 | 0 | 17 |
| Kazakhstan | 89 | 25.00 | 65.00 | 90.00 | 72.00 | 100.00 | 5.16 | - | 5 | 58 | 18 | 20 |
| Kenya | 233 | 1.30 | 8.00 | 87.00 | 82.00 | 100.00 | 6.35 | - | 50 | 44 | 0 | 7 |
| Kiribati | 551 | 5.20 | 0.00 | 87.00 | - | - | 6.99 | - | 14 | 66 | 0 | 20 |
| Republic of Korea | 80 | 3.70 | 45.00 | 81.00 | 59.00 | 100.00 | 8.60 | - | 38 | 41 | 13 | 8 |
| Kyrgyzstan | 144 | 32.00 | 33.00 | 84.00 | 57.00 | 100.00 | 4.27 | - | 4 | 56 | 10 | 30 |
| Lao People’s Democratic Republic | 182 | 5.20 | 36.00 | 86.00 | 71.00 | 100.00 | 7.36 | - | 21 | 59 | 8 | 12 |
| Lesotho | 788 | 4.80 | 21.00 | 70.00 | 63.00 | 65.36 | 12.01 | - | 29 | 33 | 1 | 37 |
| Liberia | 308 | 2.80 | <1 | 74.00 | - | - | 4.12 | - | 52 | 31 | 0 | 17 |
| Lithuania | 56 | 12.00 | 76.00 | 81.00 | 40.00 | 100.00 | 10.76 | - | 13 | 26 | 50 | 11 |
| Madagascar | 236 | 0.49 | - | 83.00 | 64.00 | 17.07 | 3.92 | - | 82 | 14 | 0 | 4 |
| Malawi | 193 | 0.75 | <1 | 85.00 | 53.00 | 69.89 | 8.69 | - | 62 | 34 | 0 | 4 |
| Malaysia | 89 | 1.50 | 44.00 | 78.00 | 62.00 | 38.12 | 9.24 | - | 61 | 32 | 2 | 5 |
| Maldives | 53 | 2.60 | 24.00 | 37.00 | - | 0.00 | 3.97 | - | 77 | 16 | 0 | 7 |
| Mali | 57 | 2.80 | 2.00 | 73.00 | 42.00 | 100.00 | 8.09 | - | 55 | 38 | 0 | 6 |
| Marshall Islands | 344 | 0.00 | 20.00 | 86.00 | 100.00 | 100.00 | 7.89 | - | 100 | 0 | 0 | 0 |
| Mauritania | 107 | 2.80 | 12.00 | 70.00 | 43.00 | 54.17 | 2.89 | - | 61 | 28 | 1 | 10 |
| Micronesia | 124 | 5.20 | <1 | 94.00 | - | 100.00 | 3.61 | - | 7 | 87 | 0 | 6 |
| Moldova | 152 | 32.00 | 56.00 | 79.00 | 57.00 | 97.89 | 11.39 | - | 4 | 45 | 20 | 30 |
| Mongolia | 428 | 2.20 | 23.00 | 86.00 | 56.00 | 100.00 | 2.16 | - | 45 | 35 | 5 | 15 |
| Morocco | 107 | 1.00 | - | 86.00 | 42.00 | 100.00 | 2.08 | - | 68 | 28 | 0 | 4 |
| Mozambique | 551 | 3.70 | 17.00 | 89.00 | 52.00 | 100.00 | 6.12 | - | 19 | 58 | 5 | 18 |
| Myanmar | 365 | 5.10 | 7.00 | 87.00 | 83.00 | 79.02 | 4.71 | - | 21 | 61 | 0 | 18 |
| Namibia | 489 | 5.00 | 9.00 | 87.00 | 74.00 | 94.83 | 6.33 | - | 18 | 61 | 0 | 21 |
| Nauru | 113 | 2.30 | 0.00 | 100.00 | - | - | 0.00 | - | 0 | 98 | 0 | 2 |
| Nepal | 156 | 2.20 | 12.00 | 92.00 | 71.00 | 84.04 | 2.68 | - | 34 | 59 | 1 | 7 |
| Nicaragua | 51 | 0.94 | 19.00 | 85.00 | 78.00 | 80.56 | 5.01 | - | 67 | 30 | 1 | 2 |
| Niger | 95 | 2.80 | <1 | 79.00 | 81.00 | 75.56 | 6.68 | - | 47 | 45 | 0 | 8 |
| Nigeria | 322 | 4.30 | 40.00 | 87.00 | 77.00 | 52.86 | 5.44 | - | 26 | 57 | 1 | 17 |
| Northern Mariana Islands | 58 | 5.30 | 52.00 | 62.00 | - | - | 15.38 | - | 45 | 36 | 10 | 10 |
| Pakistan | 270 | 4.20 | 1.00 | 93.00 | 69.00 | 83.46 | 1.44 | - | 18 | 70 | 0 | 12 |
| Palau | 76 | 0.00 | 57.00 | 57.00 | - | - | 14.29 | - | 100 | 0 | 0 | 0 |
| Panama | 50 | 3.00 | 21.00 | 79.00 | 50.00 | 100.00 | 8.36 | - | 43 | 46 | 6 | 5 |
| Papua New Guinea | 432 | 3.40 | - | 70.00 | - | 88.58 | 3.53 | - | 46 | 30 | 1 | 23 |
| Peru | 119 | 5.90 | 70.00 | 87.00 | 55.00 | 100.00 | 3.54 | - | 27 | 29 | 31 | 12 |
| Philippines | 322 | 2.60 | 1.00 | 92.00 | 49.00 | 100.00 | 2.23 | - | 26 | 64 | 0 | 10 |
| Republic of Korea | 80 | 3.70 | 45.00 | 81.00 | 59.00 | 100.00 | 8.60 | - | 38 | 41 | 13 | 8 |
| Republic of Moldova | 152 | 32.00 | 56.00 | 79.00 | 57.00 | 97.89 | 11.39 | - | 4 | 45 | 20 | 30 |
| Romania | 84 | 3.00 | 48.00 | 85.00 | 41.00 | 100.00 | 7.42 | - | 36 | 38 | 20 | 6 |
| Russian Federation | 80 | 22.00 | 38.00 | 69.00 | 48.00 | 100.00 | 9.54 | - | 12 | 50 | 15 | 22 |
| Rwanda | 56 | 1.50 | 26.00 | 86.00 | 81.00 | 98.94 | 7.80 | - | 52 | 45 | 0 | 3 |
| Sao Tome and Principe | 97 | 2.80 | 1.00 | 74.00 | 83.00 | - | 12.75 | - | 45 | 47 | 0 | 7 |
| Senegal | 139 | 0.90 | 44.00 | 87.00 | - | - | 4.38 | - | 69 | 23 | 4 | 4 |
| Solomon Islands | 89 | 5.20 | 14.00 | 91.00 | - | 100.00 | 4.64 | - | 13 | 76 | 5 | 6 |
| Somalia | 274 | 8.70 | 0.00 | 86.00 | 77.00 | 98.67 | 4.32 | - | 14 | 66 | 0 | 20 |
| South Africa | 834 | 3.50 | 65.00 | 78.00 | 48.00 | 63.87 | 6.71 | - | 36 | 23 | 7 | 33 |
| South Sudan | 146 | 3.20 | 7.00 | 71.00 | - | 0.00 | 4.36 | - | 53 | 35 | 0 | 12 |
| Sri Lanka | 65 | 0.54 | 13.00 | 84.00 | 50.00 | 86.67 | 6.82 | - | 78 | 19 | 1 | 2 |
| Sudan | 88 | 2.80 | <1 | 82.00 | 64.00 | 50.66 | 3.19 | - | 47 | 45 | 0 | 7 |
| Swaziland | 565 | 8.00 | 31.00 | 78.00 | 60.00 | 100.00 | 10.21 | - | 18 | 43 | 5 | 34 |
| Tajikistan | 87 | 14.00 | 50.00 | 89.00 | 60.00 | 94.22 | 4.68 | - | 9 | 60 | 18 | 14 |
| Tanzania | 306 | 1.30 | 13.00 | 90.00 | 68.00 | 69.10 | 5.76 | - | 40 | 52 | 1 | 7 |
| Thailand | 172 | 2.20 | 10.00 | 80.00 | - | 100.00 | 7.37 | - | 51 | 38 | 2 | 9 |
| Timor-Leste | 498 | 2.60 | 5.00 | 84.00 | 50.00 | 100.00 | 2.52 | - | 43 | 37 | 1 | 19 |
| Togo | 52 | 2.80 | 0.00 | 88.00 | 56.00 | 0.00 | 6.46 | - | 28 | 68 | 0 | 4 |
| Tuvalu | 232 | 5.20 | 5.00 | 47.00 | - | - | 33.33 | - | 33 | 47 | 1 | 19 |
| Uganda | 202 | 1.60 | 8.00 | 75.00 | 73.00 | 100.00 | 7.52 | - | 65 | 26 | 0 | 9 |
| Ukraine | 91 | 25.00 | 62.00 | 72.00 | 39.00 | 100.00 | 10.42 | - | 9 | 33 | 33 | 24 |
| Uzbekistan | 79 | 24.00 | 28.00 | 87.00 | 53.00 | 100.00 | 4.60 | - | 5 | 66 | 11 | 18 |
| Vanuatu | 63 | 0.00 | 0.00 | 87.00 | - | - | 5.13 | - | 100 | 0 | 0 | 0 |
| Viet Nam | 137 | 4.10 | 8.00 | 91.00 | 69.00 | 81.90 | 2.51 | - | 21 | 69 | 1 | 8 |
| Zambia | 391 | 1.10 | <1 | 85.00 | 33.00 | 50.51 | 5.43 | - | 59 | 32 | 0 | 9 |
| Zimbabwe | 242 | 3.20 | - | 81.00 | 59.00 | 92.52 | 8.99 | - | 37 | 46 | 3 | 13 |

^i^95% Confidence Intervals as reported in the WHO TB report 2016. ^ii^Confidence Intervals not available. The ranges are used for the Monte-Carlo method. Definitions of model parameters: **a.** Rate of MDR/RR-TB in new cases; **b.** DST coverage; **c.** treatment success rate for new DS-TB cases; **d.** treatment success rate for new MDR/RR-TB cases; **h.** proportion of notified MDR/RR-TB cases that start on second-line regimen; **m.** death rate during treatment for DS-TB; **k.** death rate during treatment for MDR/RR-TB.

See the main text for a full description of the parameters. Estimates come from the country profiles available on the WHO website (*http://www.who.int/tb/country/data/profiles/en/*). For estimates appearing as ‘<1’ on the WHO website, we used a value of 0.5 in the simulations.

Parameter values for *e* and *f* are assumed not to change according to the area. Here below are the values used for these two parameters in every region/country.

| Parameter | Description | Value (range) | Source |
| --- | --- | --- | --- |
| *e* | Treatment success rate for an MDR/RR-TB patient treated as a DS-TB patient (%) | 10 (0-20) | [^3-5^](#_ENREF_3) |
| *f* | Risk of drug resistance amplification for a DS-TB patient failing therapy (%) | 15 (10-20) | [^6-8^](#_ENREF_6) |

**4 Appendix references**

1. Wang JY, Lee LN, Lai HC, et al. Prediction of the tuberculosis reinfection proportion from the local incidence. The Journal of infectious diseases 2007;**196**(2):281-8.

2. WHO. Global Tuberculosis Report 2016: World Health Organization, 2016.

3. Bastos ML, Hussain H, Weyer K, et al. Treatment outcomes of patients with multidrug-resistant and extensively drug-resistant tuberculosis according to drug susceptibility testing to first- and second-line drugs: an individual patient data meta-analysis. Clin Infect Dis 2014;**59**(10):1364-74.

4. Millington KA, Gooding S, Hinks TS, et al. Mycobacterium tuberculosis-specific cellular immune profiles suggest bacillary persistence decades after spontaneous cure in untreated tuberculosis. J Infect Dis 2010;**202**(11):1685-9.

5. Tiemersma EW, van der Werf MJ, Borgdorff MW, et al. Natural history of tuberculosis: duration and fatality of untreated pulmonary tuberculosis in HIV negative patients: a systematic review. PLoS One 2011;**6**(4):e17601.

6. Bonnet M, Pardini M, Meacci F, et al. Treatment of tuberculosis in a region with high drug resistance: outcomes, drug resistance amplification and re-infection. PLoS One 2011;**6**(8):e23081.

7. Cox HS, Niemann S, Ismailov G, et al. Risk of acquired drug resistance during short-course directly observed treatment of tuberculosis in an area with high levels of drug resistance. Clin Infect Dis 2007;**44**(11):1421-7.

8. Dye C, Espinal MA. Will tuberculosis become resistant to all antibiotics? Proc Biol Sci 2001;**268**(1462):45-52.
